# ‘We knew it was a totally at random thing’: parents’ experiences of being part of a neonatal trial

**DOI:** 10.1186/s13063-017-2112-3

**Published:** 2017-08-01

**Authors:** Merryl Harvey, Phumza Nongena, David Edwards, Maggie Redshaw

**Affiliations:** 1 Centre for the Developing Brain, Division of Imaging and Biomedical Engineering, King’s College, London, UK; 2 National Perinatal Epidemiology Unit, Nuffield Department of Population Health, University of Oxford, Old Road, Oxford, OX3 7LF UK; 3 Faculty of Health, Education and Life Sciences, Birmingham City University, Birmingham, UK; 4 Department of Paediatrics, Walter Sisulu University, Cecelia Makiwane Hospital, Mdantsane, East London South Africa

**Keywords:** Parents, Neonatal trial, MRI, Ultrasound scan, Preterm, Trial recruitment

## Abstract

**Background:**

Studies exploring parents’ trial experiences generally relate to their understanding of the consent process and the development of researcher strategies to facilitate recruitment and retention. The aim was to better understand parents’ experience of being part of a trial at the time and their perceptions of trial participation in retrospect.

**Methods:**

Data were collected in a number of ways: from recorded discussions between parents and clinicians about the MRI or ultrasound, in open-text responses to questionnaires and in qualitative interviews at 1 and 2 years after participation. Thematic analysis was undertaken using NVivo10.

**Results:**

Key themes identified were ‘deciding to take part’, with subthemes associated with ‘benefitting self’, ‘benefitting others’ and ‘being prepared’; ‘the randomisation process’ with subthemes relating to ‘acceptance’ and ‘understanding’ and ‘actual engagement’ with subthemes of ‘practicalities’ and ‘care from responsive staff’.

**Conclusion:**

Parents’ perspectives on the trial and the processes and information received reflect their understanding and experience of the trial and the value of parent-friendly information-giving about participation, randomisation and follow-up. The practical and logistical points raised confirm the key issues and parents’ need for sensitive care and support in the course of a trial. Looking back, almost all parents were positive about their experience and felt that the family had benefitted from participation in the trial and follow-up studies, even when the developmental outcomes were poor.

**Trial registration:**

ClinicalTrials.gov, ID: NCT01049594. https://clinicaltrials.gov/ct2/show/NCT01049594. Registered on 13 January 2010.

EudraCT: EudraCT: 2009-011602-42. https://www.clinicaltrialsregister.eu/.

## Background

The complexity of studies involving children and the need for proxy consent, usually given by the parents, provides a unique set of circumstances in which to explore perceptions of research and trial participation [1]. Parents seem to welcome being asked if their child could participate in a study even if, for whatever reason, they decide to decline [1, 2]. There are studies which specifically investigated parental motivates to consenting their baby or child to taking part in a study. The most common explanations are personal benefit for the child or family and altruistic reasons [3–5]. Researchers have also looked at the views of parents on specific aspects of trials; for example, deferred consent in neonatal and paediatric clinical trials with treatment that was considered emergency, using both quantitative and qualitative methods [6–8].

Studies exploring parents’ experiences and perceptions of their child being part of a research study generally relate to their recollection and understanding of the consent process [9–12], the exploration and development of strategies to facilitate recruitment and retention [1] and waiving consent in a trial [13]. Evidence, based on interviews or focus groups suggests that the information that parents value most when deciding about their child’s participation in research is practical information about what will be involved and when this will take place [4]. Other factors influencing parental decision-making include their own knowledge and experience, their child’s health status, staff attitudes to research and the way that staff communicate with them [3].

Although some studies have investigated parents’ longer-term perspectives on their child being part of a trial [4], these are few in number. The present study was part of a programme of work that involved a clinical trial, with qualitative components that focussed on parental perceptions and experience over the whole course of their participation in the planned research programme through to outcomes for their children at 2 years [14]. The aim was better to understand parents’ experience at the time, in close proximity to the intervention and their perceptions of trial participation in the longer term.

## Methods

The ePrime study, involved a trial of information-giving to parents following cerebral magnetic resonance imaging (MRI) and ultrasound (US) scans undertaken at term of babies who had been born before 33 weeks’ gestation. We carried out a parallel-group randomised controlled trial with 1:1 allocation that compared the effect of prognostic information derived from either MRI or ultrasound on parental anxiety and coping, health costs, and health-related quality of life; and in a nested diagnostic evaluation with blinded assessment compared the precision of the two types of scan. Thus, in the randomised controlled trial (RCT) we tested whether information from MRI improved infant care and family wellbeing compared to information from cerebral ultrasound. Parents received either the MRI or ultrasound scan result and prognostic information based upon that particular scan. Families were subsequently followed up and a full developmental assessment of the child was carried out at 20–24 months’ corrected age.

Babies were recruited to the ePrime study while being cared for in one of 13 neonatal units in the London area. Written informed consent from parents was obtained at the recruitment site and affirmed at the scanning appointment at the specialist centre. Data measuring and exploring the impact on parents’ of the scan result were collected over 2 years and the data presented in this secondary analysis were drawn from three study components:

1. Audio-recordings were made at the time of discussions between parents and clinicians about the baby’s MRI or ultrasound result with parental agreement and consent. These discussions took place immediately following the scan at term. The recordings were fully transcribed and analysed thematically using NVivo10 [15]. While the audio-recordings of the clinician-parent discussions were primarily undertaken to capture the ways in which medical staff gave information based on the baby’s scan findings and prognosis [16, 17] the discussion also involved parents’ reactions to allocation and more generally to participation in the trial. The recordings were made on a subset of 60 clinician-parent discussions targeting three time periods during the first, second and third years of the study, carried out consecutively. Analysis was based on transcripts of 36 of these interviews, saturation having been reached
2. Parent-completed questionnaires, containing standard measures and general questions giving them the opportunity to respond in their own words, were sent to all parents following the term scanning appointment. The questionnaires were administered at three time points: shortly after the term scan visit, 1 year post scan and immediately prior to the developmental assessment carried out 2 years after the scan
3. Qualitative interviews with subsamples of parents were carried out by telephone around the time of their child’s first birthday and when their child had reached approximately 2 years’ corrected age. Those parents who had previously consented to interview were invited to reaffirm their consent by the research nurse when contacted. Initially, parents were randomly selected; however, towards the end of data collection purposive sampling was used to ensure diversity of this sample in terms of age, education and ethnicity. A topic guide was used by the interviewer, which included references to the trial and scanning appointment day. Efforts were made to interview both mothers and fathers. No attempt was made to interview the same parents at 1 and 2 years. The interviews were recorded and fully transcribed

Transcriptions of the parent-clinician discussions, open-text responses in the questionnaires and transcriptions of the 1- and 2-year interviews were analysed thematically with a focus on study participation using NVivo10 [15]. It became apparent in the course of analysis, when examining data from individual study components, that there were commonalities in terms of the themes relating to participation in the trial. Thus, these qualitative data were pooled and analysed thematically in this secondary analysis [18]. Extracts of the data relating to trial participation were coded and the codes were organised into themes and subthemes. New codes were generated when the data appeared to capture something different. The coding framework was then reviewed and revised until the final structure was agreed by MR and MH, leading to the identification of key themes.

## Results

A total of 434 families with 510 babies were recruited and participated in the trial, of which 210 were in the MRI arm and 214 in the US arm of the trial, 36.2% of those eligible [14]. All the babies were born before 33 weeks’ gestation (mean gestational age of 30 weeks and a mean birth weight of 1305 g.)

Audio-recordings of clinician-parent interviews at around term involving 34 mothers, 17 fathers and 1 grandmother, related to 43 babies (including five sets of twins and one set of triplets) were analysed. Most families (80% of the 434 participating in the main study), agreed to participate in the audio-recorded discussions. A total of 60 recordings were made and a subset of 36 of these carried out by different clinicians, evenly distributed over the three different years of the study, were analysed. The discussions reflected information-giving and parent’s understanding and perceptions of the trial.

In the 434 families more mothers than fathers completed the questionnaires: 84% of mothers and 79% of fathers, shortly after the scans were carried out; 82% and 62%, respectively, at 1 year and 97% and 82%, respectively, at 2 years. Of the parents who completed questionnaires, over 94% responded to an open-text question in one or more of the questionnaires.

At 1 year, 30 parents (25 mothers, 5 fathers) were interviewed by telephone. The interviews related to 35 babies (including four sets of twins and one set of triplets). During these interviews parents talked about why they had agreed to take part in the study and recalled the scanning visit. This led to discussion about their perceptions of the study and the way in which it was conducted.

At 2 years, 30 parents (21 mothers, 9 fathers) were interviewed by telephone several weeks after the 20–24 months’ corrected age developmental assessment. The interviews related to 31 babies (including four sets of twins). During these interviews, parents recalled the recent developmental assessment and the discussion led them to reflect upon the totality of their ‘ePrime experience,’ and their reasons for taking part in the study. None of the parents who took part in the 2-year interview had participated in the 1-year interview.

This secondary analysis led to the identification of three key themes about experience of the trial, which include a number of subthemes (Table 1).

**Table 1:** Key themes and subthemes relating to participation in trial and follow-up

Key themes	Subthemes
Deciding to take part	Benefitting others:
	• Giving something back
	• Helping other babies and families
	• Improving the provision of care
	Benefitting self, baby and family:
	• Feeling valued
	• Access to information and knowledge
	• Hoping for magnetic resonance imaging (MRI) result
	• Support from research team
	• Prompt and early diagnosis
	Being prepared:
	• Thinking through what is involved and what might happen
	• Inadequate information about the study
The randomisation process	Accepting the process:
	• What we would have liked
	Understanding randomisation:
	• An equal chance
	• Only one result
Actual engagement	Practicalities:
	• The way the study was conducted
	• Impact on parents
	Care from responsive staff:
	• Being looked after
	• Needs recognised

Evidence for all of the trial-related themes and subthemes arose from two or more of the data sources. The themes and subthemes are described with direct quotes used for illustration. Details associated with the quotations are as follows: ‘M’ denotes mother, ‘F’, father and ‘C’ clinician; ‘AR’ indicates audio-recording; ‘Q2’, ‘Q3’, and ‘Q4’indicates the questionnaire completed after the scan and at 1 and 2 years; the year-1 and year-2 interviews are indicated as ‘Y1 Int’ and ‘Y2 Int’, respectively.

### Deciding to take part

This key theme which describes the feelings and self-ascribed motivation of parents who took part in the trial. Three subthemes were identified. The first two relate to the rationale that parents put forward for consenting to their child’s participation in the study. Reciprocity was a construct underlying this theme and is reflected from two perspectives: parents framed their reasons in terms of ‘benefitting others’ and ‘benefitting self’ (the baby, themselves and the wider family). The third subtheme which arose in the context of deciding to take part was ‘being prepared’ which concerns their reflections on their own level of understanding at the time of making the decision to participate.

#### Benefitting others

Parents were explicit about valuing what they had received in terms of health care, particularly the neonatal care that their baby had received. They gave altruistic reasons in principle for agreeing to involvement in the study: they wished to help other babies and families and referred to wanting to *‘give something back, after all the help that you get during the process of having a premature baby’*. However, they were less specific about how this might work. Nevertheless, all parents were pleased to *‘be part of it [the study]’*, grateful for the care their family had received and felt their involvement in the research had the potential to *‘help other people as well’* and improve the delivery of neonatal care in the future. At the same time they emphasised the smallness of their role and acknowledged the incremental way in which it might help. The references to giving back continued over the 2-year period that followed recruitment:

M: ‘Oh we want to do it anyway, because they were so good.’

C: ‘Well, thank you, thank you for that. We’re very grateful, for that.’

M: ‘If it helps others…’

F: ‘I mean, I was a bit dubious about doing it, wasn’t I? But you know, they were really good in X [hospital]. Well you know, if a tiny percent helps anyone else.’

M: ‘Exactly.’ AR 305

‘I’ve always thought it was a good idea for the girls to get involved in stuff like this, you know, studies and that. So the one thing that’s been stuck in my mind is that this is their time to help.’ Y1 Int M1266-1279

‘I think we were in that situation where we felt it would be very easy just to say “no” and let’s just not do it. But at some point, you know, if things are to improve, someone has to say, “let’s do this”. We decided that this had happened to us and actually someone might benefit down the line, if we actually do something.’ Y1 Int M3239

‘I’m glad to be part of the research programme – if not for me – to help others.’ Q2 M3095

‘I think, you know, it’s, err, nice to feel that we can contribute in a very small way, but just towards, you know,…the amazing things that can be done for premature babies now.’ Y2 Int M1776-1769

#### Benefitting self – should be benefitting self, baby and family

Most parents gave altruistic reasons for taking part in the research in principle; they specifically referred to the potential benefits to their baby, themselves and their wider family. They particularly felt that the opportunity for this more detailed scan could provide them with knowledge and additional information about their baby’s prognosis:

‘Well it’s great to be able to be part of something like this because either way you get to go away with more information… And you can move forward that way.’ AR M1636

‘Simply I think one of the main reasons, the first main reason is the fact that it gave us the opportunity of possibly finding out a bit more about the situation.’ Y1 Int F1130

Although not directly asked, most parents commented that they hoped to be given the MRI findings at the scanning appointment and the possibility of that and the resulting information was a potential gain. This was recognised in close proximity to the scanning and also reflected on over a longer time period:

C: ‘The results you’re going to get are of the MRI.’

M: ‘Oh, fantastic.’

F: ‘Great.’

M: ‘I think we were hoping for that actually, yea.’ AR 1266-1279

‘I think the possibility of getting the MRI helped us to decide.’ Yr1 Int M3239

‘We did it hoping to get the MRI result.’ Yr 2 F4845

Similarly, parents agreed to take part in the study because of the developmental assessment which could provide further information about their child’s progress and further prognosis. They were explicit about the ongoing contact provided by the research team during the study being another reason why they agreed to participate. At the same time parents could feel valued at being asked to take part in the study and some alluded to their own feelings of self-worth in deciding to participate:

‘I think I took part because you don’t know when you have a premature baby. You don’t know what’s wrong with them. You’re never going to find out, you know, I mean obviously they give you information why this or why that, in my circumstance it was myself that was ill and that caused 2583 to come 3 months early. But other people, babies just don’t grow and they have to come out. So I think the benefit of it and knowing, you know, this is good. You know, you have your babies normally at full term and you go home within 3, 4 hours. But here I was thinking, well 2583 was in hospital a long time. So it’s nice to know and also to be quite honest, nice to have the support there after he was brought home. You know, they ring me and we have a chat, he got his first little birthday card from them. For me that’s something that I want to keep for him when he grows up.’ Yr 1 Int M2583

‘I do remember that was another reason why we were happy to take part in this study, because we will be coming in with the children when they’re 2 years old and they’ll be assessed and it will be really good for us to know where they’re up to when they’re at that age.’ Yr 1 Int M2047-2059

‘I think, you know, for us one of the reasons why we decided to be part of the ePrime study was because, you know, it wasn’t just…, it wasn’t so much to do with the actual brain structure or anything like that, it was all to do with her developmental needs.’ Yr 2 Int M4063

‘It was wonderful to be part of it, we’re just glad we were asked and that we could be part of it.’ Yr 2 Int F1910

#### Being prepared

Within this subtheme parents talked about whether or not they had been adequately prepared for what the scanning actually involved and the results that they might receive in participating in the trial. For some, it was only in retrospect that they realised that they had been poorly prepared. This was either because of their own lack of understanding about the study requirements, not thinking through what the study could involve emotionally or practically, or the implications for them of their child’s participation. Insufficient explanations or limited information from the research team about the scans was also felt to have contributed:

‘I found it very difficult returning to the neonatal unit for the MRI scan etc.’ Q2 M3262

‘The sedation, I don’t think that had properly been explained because they had to give it in a certain way, it was oral sedation and that was quite distressing.’ Yr 1 Int M3088

‘I was a bit concerned about her having the MRI when we actually got there and saw the room and saw the machine. She just seemed so little.’ Yr 1 Int M2345

‘I just remember being there, I just remember that it was intense and it felt slightly intimidating because it seemed so dramatic.’ Yr 2 Int F1910

Although their babies had undergone scans while in the neonatal unit, several parents said that they were quite unprepared for the possibility of being given an abnormal result at the scanning visit around term when US and MRI scans were carried out:

‘Was very worried about the results when the scans were being done – hadn’t really thought before the scanning day that may have been given bad news.’ Q2 F7536

‘I did get a little upset at the MRI scan as I didn’t realise that the tests might find something wrong with my son. I wasn’t prepared mentally to find out any bad news.’ Q2 M3321

The same mother reiterated the point later when her child had reached the age of 2 years:

‘I wasn’t aware what they were looking for with the scan… I wasn’t prepared for this.’ Q4 M3321

I do think though at the time, we didn’t really prepare ourselves for how we would feel when we got the results… We thought about being helpful, you know the good intention of the project, but I don’t think we’d really thought through how it was going to affect us, in terms of how are we going to then deal with whatever we’re told.’ Y1 Int M2345

### The randomisation process

The second key theme focusses on issues relating to acceptance of the randomisation process, and whether or not parents appreciated what randomisation meant for them and their baby.

#### Accepting the process

Most parents understood the structure of the trial as described to them and recognised the difference between their wishes and what the study design required. Parents who referred to randomisation generally apprehended that there was an equal chance of them being given the MRI or US result and why randomisation was undertaken:

‘We did it hoping to get the MRI result, but we knew there was a 50:50 chance.’ Yr 2 Int F4845

‘Would have liked MRI result but understand randomisation process.’ Q2 M5977

‘Yes, it was the ultrasound and not the other one, the MRI. I think we probably would have preferred the MRI because we thought it would be more in detail and more, but we knew it was a totally at random thing.’ Yr1 Int M3239

While most had accepted the notion of randomisation, at the same time some acknowledged they would have preferred to be in the MRI arm of the trial:

‘I would love to know the results of the MRI scan! I realise, however, that this would negate the purpose of the study!’ Q2 M1358

However, a few parents *‘would have liked both results’* and on the scanning day they were not prepared for only one result, though they had been told that at the 2-year follow-up they would be given the result of the ‘other’ scan:

‘Would have liked result of “other” scan – didn’t realise wouldn’t get both results.’ Q2 M5301

The qualitative data did show that a few parents had not understood what was implicit in the randomisation process and that this was integral to the trial:

‘The selection on which result is get is unfair.’ Q4 M2675

‘I didn’t understand why the results were split between MRI and ultrasound. I would have liked both results.’ Q2 F4362

Some parents felt mixed about having the results and the role of the randomisation process which may have meant only having some of the information available:

‘We were very upset for a few weeks following the scan and results. We found it difficult to come to terms with the possibility of problems down the line.’ Q2 M1017 (MRI result received)

‘It was reassuring that the scan did not pick up any problems, but as it is not conclusive, it is hard to fully relax due to the results.’ Q4 M1757 (US result received)

‘I would have liked more information about the type of outcomes and what it meant/could mean, that is what could an MRI determine for example, what empirical evidence is there about imaging and future cognitive impairment etc.’ Q2 M7427 (MRI result received)

### Actual engagement with the study

Within this key theme, two subthemes were identified that arose from parent’s reflections on what the study really involved. The first focusses on the ‘practicalities’ of the study processes and the second on ‘care from responsive staff’.

#### Practicalities

This subtheme arises from parents’ comments about the way in which the study was conducted. There was a particular focus on practical and logistical issues and the ways in which these impacted on their child, themselves and the family. Many of the comments related to the scanning appointment; the care that they and their babies received, the facilities available and managing the scanning of twins and triplets on the same day. While the scans were seen as valuable, the clinical environment and necessary procedures could be perceived as uncomfortable and distressing. Having finally taken their preterm babies home, several parents described the challenge of bringing them back to a hospital for the research study so soon after discharge. Some parents had clearly been conflicted on this point and referred to practical issues such as a lack of space and facilities for suitable for siblings, being back in hospital and negative issues relating to feeding and the use of sedation. Feeling that they were doing the right thing was moderated by some of the difficulties experienced at this time:

Appointments that involved twins and triplets were longer and more tiring:

‘Yes, it was a pretty tough day because it was a bit full-on, so, I can’t remember which one of them actually was a bit fractious that day. But it was a long day.’ Y1 Int F2047-2059

‘Helpful to have full facilities for baby care. Generally a long day with twins but easier to do together than on separate days.’ Q4 M6470-6487

Most parents, though they may have been worried at the start of the discussion, did not receive abnormal results and on reflecting back later indicated that they enjoyed the scanning day and that they had been made to feel welcome, comfortable and relaxed: *‘You feel sort of at home here’; ‘Everything was fine today. I’m normally quite nervous’; ‘We’ve had a good day out’;* and *‘it felt like it was done very well indeed’*:

‘I would just like to add that throughout the day we were well looked after which made us feel at ease whatever the outcome of the test results were going to show.’ Yr 1 Int M2753

Many commented positively about the conduct of the study, particularly what was seen as the later follow-up of their child’s development, valuing the arrangements and the way in which they had been *‘looked after’* in the context of the *‘assessment’* or *‘test’* that was carried out:

‘Thanks for looking after us so well on the assessment day; this was the first time we has been out all day and in itself was a great confidence boosting exercise with the safety net of being in a hospital!’ Q2 M1447-1459

#### Care from responsive staff

The importance of a responsive and professional research team was recognised and the individualised nature of the care that parents felt they received was emphasised in the text giving rise to this subtheme capturing parents’ thoughts about the study team carrying out the trial. They referred to a need to be treated *‘sympathetically and respectfully’* and staff who had *‘consideration and patience with us… bearing with us’*:

‘Staff [were] very supportive when I got emotional [at the scanning appointment].’ Q4 M2663

‘So when we came there the first time [scanning appointment]… the people who have been with us have been very sensitive and very, very good, so it’s been a very good experience for us.’ Yr 2 Int M1804

While most were parents were grateful to the research nurses and physicians for the way in which the scans were carried, not all were positive. Critical comments generally related to what parents saw as a lack of knowledge or sensitivity. This particularly related to feeling their role as a parent was being usurped or negated:

‘The nurses kept taking my daughter off me [at the scanning appointment], i.e. to undress her, etc. Being back in the neonatal is difficult enough without nurses trying to do everything as well.’ Q2 M3262

‘I think it’s crucial that staff make sure parents don’t feel excluded from their baby’s care. When our baby had her assessment [scanning] visit, she was taken away, without our permission, because she started crying when the doctor was speaking to us. This was apparently to help us focus, but it resulted in quite the opposite effect. I was also made to feel in the way when I attempted to comfort her during her head scan.’ Q2 M3438

‘One of my baby’s earplugs came off over and over again… I let them know… Then X [health care professional] told me to leave the MRI room. …Her attitude was extremely aggressive, and I felt as if she was saying I was an idiot who didn’t listen to her. This made me so upset and angry. I considered calling off the test. I volunteered to bring my babies to the test and saw them not handling my babies with confidence.’ Q2 M1515-1521

## Discussion

This paper addresses the need identified by Shilling et al. [1] to inform clinicians and practitioners what parents think about participating in trial-based research. The parts played by altruism and self-interest in ‘deciding to take part’ in the trial were identified in the analysis and are evident in the subthemes of ‘benefitting self’ and ‘benefitting others’. The subtheme ‘being prepared’ highlights the key need for participants to have access to good quality information, to be well informed and prepared for what might be learned as a consequence of the trial. ‘Actual engagement’ reflects ‘the practicalities’, that is the realities of participating in the trial for parents and their needs in terms of sensitive and ‘care from responsible staff’.

The themes identified in this study echo some aspects of other studies in the academic literature have identified [19, 20]. In a systematic review of studies on consent to clinical trials with pre-term or sick neonates, the basic motivations of parents in agreeing to research [20) were fairly consistent across multiple studies [4]. Points of difference are likely to be related to study design, timing and the nature of the research question asked.

In the trial on which this study was based, the intervention involved significant diagnostic and prognostic information-giving, but did not, as with some clinical trials, take place in an urgent or emergency context. Neither did it involve a clinical intervention such as medication given in a placebo-controlled trial [21, 22], though it did involve outcome assessed at 20–24 months’ corrected age. Relatively well preterm babies and mothers were scanned having been discharged from hospital [14]. Knowing more about their baby’s condition and the possibilities for the longer term was perceived as a particular benefit of participation for parents taking part in this trial. The prognostic information provided following the scanning appointment and the longer-term follow-up were personal drivers for participation and for some parents contributed to empowerment. The small babies and the mismatch in size with the MRI equipment, together with the relief experienced when results were in the normal range underpinned some parents’ views about the trial experience. The painful emotional responses of some mothers to some of the interactions with the health professionals on the scanning day may reflect echoes of their neonatal experience, their continued vulnerability after their baby had been discharged home and their strong sense of the need to naturally protect and care for their babies in the clinical environment [23, 24].

Other researchers have noted that benefits are not always tangible. Increasing self-esteem, pride in participation [25] and feeling valued were important to parents in this study as was also found in non-trial studies [5]. More broadly, the study parents valued the opportunity to participate and described appreciating being part of something bigger, in a continuing way [1].

The study adds to the evidence about parents’ experience of trials. Engaging them in research and providing sufficient and appropriate information for them to be well informed, while balancing their information and emotional needs is not easy and checks need to be made on their understanding both at the time of the recruitment and consent processes, and following the intervention [17]. The demands and expectations placed on parents need to be thought through. Most participate willingly, but there is an impact and a potential emotional burden and staff should not underestimate parents’ emotional as well as information needs in trial participation, particularly in bringing their baby or babies back to the hospital environment. A challenge for those taking consent is to ensure that parents understand all aspects of the study and the implications without overloading them with lengthy, complex information [26]. Ensuring that the randomisation process is understood by all participating parents is critical in avoiding misunderstandings about this necessary aspect of study design and its implications for care or treatment [21, 22, 27].

### Strengths and limitations

The triangulation of data using the different sources available in this study, that is parents’ reflection on their experiences (interviews and questionnaires) and communication with clinicians (audio-recordings) at the start of the trial is a strength, as is the number and diversity of parents involved [1]. The study also captured the views and experiences of mothers and fathers. As with other studies on this topic, a limitation may be that parents could have felt less able to make critical comments during the early stages of trial data collection due to the recency of the care that their infants had received. However, some clearly did feel able to make negative comments about the way in which the study was conducted as shown in some of their responses to the questionnaires and in the interviews. The time lag between early parts of study and later interviews and questionnaires may have affected parental recall; however, similar views were expressed across the whole period of data collection. The longer time frame, during which parents may have become less anxious, allowed them to reflect and may be considered a further strength.

### Implications for practice

The themes identified and parents’ responses suggest that there is a need for clinicians and practitioners to reflect on their practice and the ways in which they give information and interact with parents during a study. An inevitable challenge is that what is right for one family is not necessarily right for another. Recognising the parental role during studies of preterm infants and at clinic is critical from the parents’ perspective, as most will have experienced early separation and the rollercoaster of feelings common to parents following preterm birth. The goal of providing individualised care for each family, even within a trial, in addition to provision in the broader environment of neonatal and paediatric care is critical.

## Conclusion

The comments and reflections on the trial described in this paper represent an opportunity to better understand what trial participation meant to parents, both at the time and subsequently. The findings provide insights on the issues that are important for parents and the ways in which they viewed the trial design, recruitment and research process. Almost all were positive about their experiences and felt that the family had benefitted from participation. The practical and logistical points raised confirm the key issues with regard to research conducted on young babies and their parents and their need for sensitive care and support in the course of a trial.

## Abbreviations

MRI: Magnetic resonance imaging
US: Ultrasound
